# Determinants of Multimethod Contraceptive Use in a Sample of Adolescent Women Diagnosed with Psychological Disorders

**DOI:** 10.1155/2011/510239

**Published:** 2011-09-29

**Authors:** Delia L. Lang, Jessica M. Sales, Laura F. Salazar, Ralph J. DiClemente, Richard A. Crosby, Larry K. Brown, Geri R. Donenberg

**Affiliations:** ^1^Department of Behavioral Sciences and Health Education, Rollins School of Public Health, Emory University, Atlanta, GA 30322, USA; ^2^Center for AIDS Research, Emory University, GA 30322, Atlanta, USA; ^3^College of Public Health, University of Kentucky, Lexington, KY 40536-0003, USA; ^4^Department of Psychiatry, Brown University, Providence, RI 02912, USA; ^5^Department of Psychology, University of Illinois at Chicago, Chicago, IL 60607, USA

## Abstract

*Objective*. Despite recommendations for concurrent use of contraceptives and condoms to prevent unintended pregnancy and STIs, multimethod contraceptive use among women is poor. This study examined individual-, interpersonal-, and environmental-level factors that predict multimethod use among sexually active adolescent women diagnosed with psychological disorders. *Methods*. This multisite study analyzed data from 288 sexually active adolescent women who provided sociodemographic, psychosocial, and behavioral data related to birth control and condom use. *Results*. 34.7% of the participants reported multimethod use in the past three months. Controlling for empirically and theoretically relevant covariates, a multivariable logistic regression identified self-efficacy, multiple partners, pregnancy history, parental communication, parental norms about sex, and neighborhood cohesion as significant predictors of multimethod use. *Conclusions*. While continued targeted messages about multi-method contraceptive use are imperative at the individual level, an uptake in messages targeting interpersonal- and environmental-level factors such as adolescents' parents and the broader community is urgently needed.

## 1. Introduction

Sexually transmitted infections (STIs) and unintended pregnancy are two significant public health issues that continue to affect adolescent women in the US today. Current estimates by the Centers of Disease Control and Prevention (CDC) suggest that 25% of adolescent women in the US have an STI [1], a prevalence twice as high as that among adolescent men of the same age [2]. Similarly, an estimated 82% of adolescent pregnancies are unintended despite widespread accessibility of safe, effective, and affordable contraceptive technology [3]. While a national decline in unintended pregnancies among adolescent women between 1991 and 2009 has been documented, the US continues to have higher rates of unintended teen pregnancies compared to other developed nations, particularly among adolescents residing in southern states [4]. 

Data on the prevalence of STIs and unintended pregnancy among adolescent women diagnosed with psychological disorders are largely lacking, although extant research among adolescents demonstrates an association between mental illness and high-risk sexual behavior including early initiation of sexual activity, sex with multiple partners, and unprotected sex [5–11]. Results from these studies suggest that this vulnerable population is at great risk for acquiring STIs including HIV as well as experiencing unintended pregnancy. 

Adolescents living with psychological disorders are characterized by a unique profile of antecedent factors covering the spectrum of individual-, interpersonal- and environmental-level factors that contribute to their engaging in high-risk sexual behaviors and risk for STI/HIV infection. These factors include personal attributes (e.g., impulsivity, self-destructive behaviors, cognitive impairment, substance use, and poor judgment), family context (e.g., parental attitudes and behavior, parent-adolescent communication, parental monitoring, and discipline), peer and partner relationships (e.g., relationship concerns, peer influence, and partner communication), and environmental context (e.g., poverty) [12–17]. While the aforementioned studies have associated this risk profile with elevated exposure to STI/HIV infection, a similar risk profile may also result in unintended pregnancy among adolescent women seeking mental health services, although no studies to date have established this association.

Due to the prevalence of STIs and unintended pregnancies among youth, professional organizations such as the CDC, American Medical Association, American Academy of Pediatrics, and the American College of Obstetrics and Gynecology have recommended dual protection strategies toward the prevention of both STIs/HIV and unintended pregnancy [18–20]. Despite these recommendations, studies have demonstrated that dual-protection is not a commonly used strategy. For example, data from the Youth Risk Behavior For example, data from the Youth Risk Behavior Survey suggest that only 6.6% of young women surveyed used both a condom and oral contraception at their last sexual intercourse [21]. Similarly, data from the National Survey of Family Growth suggest that 8.5% of adolescent women reported the same dual-protection method of condom use and oral contraception in the previous 30 days [22]. However, among a sample of African-American adolescent women, 13.5% reported using dual protection [23]. 

Correlates of dual-protection use have been investigated by few studies showing that, among adolescent men and women between the ages of 14 and 22, younger African-American adolescents who communicated with their parents about HIV-related issues were more likely to report dual-protection use [21, 22]. Among low-income, minority, urban adolescent African-American women, those with a history of STIs who reported concerns about both pregnancy and HIV infection were also more likely to report dual-protection use [23]. Finally, in a recent study investigating correlates of dual-protection use in an array of contextual influences including individual-, interpersonal-, and community-level factors, results suggest that lower impulsivity, fearful relationship style, and fear of condom negotiation as well as higher self-esteem, social support, and partner communication self-efficacy were positively associated with dual-protection use [24]. No studies to date have investigated factors associated with dual-protection or multimethod contraceptive use among adolescent women seeking psychological services. The distinction between dual-protection and multimethod use suggests that the former category includes those who use dual methods concurrently, while the latter category includes those who use multiple contraceptive methods, but not necessarily concurrently. 

The current study examines the prevalence of multimethod use as well as individual, interpersonal, and environmental factors associated with multimethod use among a sample of urban adolescent females seeking psychological services at the time of the study.

## 2. Methods

### 2.1. Participants

This is a substudy of a larger multisite family-based randomized clinical trial designed to evaluate the efficacy of an intervention to reduce HIV risk behaviors and STIs while enhancing HIV-preventive psychosocial and structural factors among adolescents diagnosed with psychological disorders. Adolescents were eligible if they were between the ages of 13 and 18, received a clinician-based psychological diagnosis along the externalizing-internalizing spectrum of disorders, received in- or out-patient mental health treatment at one of the study recruitment sites, lived with a parent or guardian who was also willing to participate in the study, and provided informed consent. Externalizing disorders include those that manifest in one's outward behavior (e.g., conduct disorder, oppositional defiant disorder), while internalizing disorders include those that manifest in terms of one's thoughts and feelings (e.g., depression, anxiety). Adolescents diagnosed with schizophrenia and other psychotic disorders were excluded. Additionally, adolescents were excluded if they had a history of sexually aggressive behavior (i.e., perpetrators of sexual assault or molestation), were currently pregnant, were known to have tested positive for HIV, or had cognitive deficits precluding them from completing the assessment independently or participating in group activities. Participants were enrolled in the study at three recruitment sites: Brown University, Providence, Rhode Island, University of Illinois, Chicago, and Emory University, Atlanta, Georgia. Clinics and hospitals providing mental health services to adolescents served as recruitment sites. Of 1102 adolescents who met eligibility criteria, 891 (81%) agreed to participate and subsequently completed baseline assessments. Of the 891 participants, 288 (32.3%) were sexually active females who completed baseline assessment prior to randomization in the HIV trial and comprised the sample analyzed in the current study. The remaining 603 participants were either male or sexually inexperienced females and were excluded from the current analyses. The Institutional Review Boards at Brown University, University of Illinois, Chicago, and Emory University approved the study protocol.

### 2.2. Procedures

Adolescents and parents completed an audio-assisted computerized interview at baseline. The adolescent interview assessed sociodemographic characteristics, sexual behavior patterns, and psychosocial characteristics as well as psychological symptomology. The parent interview assessed sociodemographic characteristics, parent norms about sexual behavior, and variables addressing the frequency of parent-adolescent interactions such as communication about sex. On average, interviews were 90 minutes in duration for teens and parents independently.

### 2.3. Measures

**Multimethod Use**

Participants were asked to identify on a list of commonly used birth control methods the one(s) they utilized in the previous 3 months: male condom, pill, diaphragm, cervical cap, gels/creams/suppositories or foams, Depo-Provera, and other (not further identified). Those who reported condom use and at least one other birth control method were categorized as multimethod users; those who reported using no birth control and/or only one birth control method were categorized as non-multimethod users.

Participants' age, race, ethnicity, current relationship status, and history of pregnancy were collected from adolescent participants. Family income and parent marital status were collected from parent participants.

**Individual-Level Measures**

**Sex Partner Variables**

Adolescent participants were asked how many sexual partners they had in the past 3 months. Those reporting two or more partners in the past 3 months were categorized as having multiple partners. Additionally, participants were asked whether or not they were in a current relationship with a sex partner.

**Condom Use Self-Efficacy**

Adolescents' self-efficacy for condom use was assessed using a 13-item scale that included items such as “How confident do you feel that you could use a condom when your partner doesn't want to?” or “How confident do you feel that you could use a condom with a new partner?” [25]. Answer options ranged from 1 (very sure I could) to 4 (very sure I could not); these responses were reverse coded such that higher scores on the condom use self-efficacy scale suggest higher perceived self-efficacy for condom use. Responses may range from 13 to 52. Cronbach's alpha reliability for this scale was  .93, indicating a high level of internal consistency.

**Interpersonal-Level Measures**

**Sexual Communication with Parent**

The Miller Sexual Communication scale was used to assess the frequency of communication between parents and adolescents with regard to 6 areas including initiation of sex, contraception, condom use, transmission of HIV/AIDS, peer pressure regarding sexual behavior, and selection of sexual partner [26, 27]. Parents were asked to indicate how frequently these topics were discussed with their adolescent daughter in the past 3 months with answer options including 1 (never), 2 (once), 3 (a few times), and 4 (more than 3 times). Parent responses to the six areas of communication were summed for a total communication frequency score with higher scores suggesting more frequent communication. Responses may range from 6 to 24. Cronbach's alpha reliability for this scale was  .85, indicating a high level of internal consistency.

**Parental Norms about Sex**

Adolescents' perceptions of parents' degree of approval of the adolescent's sexual activity was assessed with this 7-item scale [28]. Sample items include “My parents think it is ok to have sex after one or two dates” and “My parents think it is ok to have sex with the person I love.” Answer options ranged from 1 (very true) to 5 (very false) with a possible range of score from 7 to 35. Cronbach's alpha reliability for this scale was  .75, indicating an adequate level of internal consistency.

**Environmental-Level Measures**

**Neighborhood Cohesion**

Neighborhood cohesion was assessed using a 6-item scale assessing whether adolescent respondents (1) visited neighbors, (2) could go to their neighbors for advice, (3) regularly talked to people in their neighborhood, (4) knew people's names, (5) felt comfortable asking to borrow things, and (6) felt comfortable asking neighbors to watch their home [29, 30]. Answer options ranged from 1 (strongly disagree) to 5 (strongly agree). Cronbach's alpha reliability for this scale was  .88, indicating a high level of internal consistency.

### 2.4. Statistical Analysis

First, descriptive analyses were conducted to obtain means, standard deviations, and proportions for relevant sociodemographic variables. Additionally, bivariate analyses consisting of chi-square and independent samples *t*-tests were performed to examine the associations between multimethod and non-multimethod use and sociodemographic variables as well as to identify potential covariates. Finally, a multivariable logistic regression model was conducted to explore whether individual-, interpersonal-, and environmental-level factors predicted multimethod use after controlling for confounders. Data were analyzed using PASW 18.

## 3. Results

A total of 288 adolescent women participated in this study with a mean age of 15.29 (SD = 1.26; range 13–18). One-third of parents (*n* = 96) reported being married, and approximately 59% (*n* = 159) of families reported an annual income of less than $30,000. Nearly 31.0% (*n* = 89) of families self-identified as Caucasian, 54.5% (*n* = 157) as African American, 12.5% (*n* = 36) as Hispanic, and 2% (*n* = 6) as other. Among parents, 18.1% (*n* = 52) had not completed high school. Mental disorders represented in this sample are as follows: oppositional defiant disorder (75.8%), ADHD (56.3%), conduct disorder (49.6%), generalized anxiety disorder (42.4%), major depression (41.3%), mania (29.7%), posttraumatic stress disorder (27.1%), and hypomania (26.9%). These rates represent dual diagnoses, therefore will not add up to 100%. Among adolescent participants, 57.6% (*n* = 166) reported having a current sex partner, and 16.2% (*n* = 46) reported a pregnancy in the past. Finally, 34.7% (*n* = 100) of adolescent participants reported multimethod use, while 50% (*n* = 144) used only one form of protection, and 15.3% (44) used no protection at all. 

Descriptive statistics as well as differences between multimethod and non-multimethod users are presented in Table 1. Of these, adolescent age, ethnicity, household income, parent marital status, and adolescent current relationship status were statistically related to multimethod use at *P* ≤ .20 level and were therefore included as covariates in the multivariable logistic regression model [31]. Although race was not associated with multimethod use at the bivariate level (*P* = .68), we controlled for this variable in our multivariable model predicting multimethod use outcome based on the prior literature suggesting that African-American adolescent women had higher rates of dual-protection use compared to other groups [23]. Bivariate associations between individual-, interpersonal-, and environmental level factors and multimethod use are presented in Table 2. 

Results of the multivariable logistic regression model are presented in Table 3. After controlling for covariates, significant predictors of multimethod use emerged among all three contextual levels of analysis. Among individual-level predictors, with each unit increase in condom use self-efficacy, adolescent women were 7% more likely to report multimethod use (*P* = .004). Additionally, participants who reported multiple sex partners were nearly three times more likely to report multimethod use compared to participants reporting a single sex partner (*P* = .011). Finally, pregnancy was a marginally significant predictor suggesting that those with a history of pregnancy were 59% less likely to report multimethod use (*P* = .052). Neither race nor ethnicity was significantly related to multimethod use. Among interpersonal-level predictors, with each unit increase in communication about sex (as reported by the parent), adolescent women were 3% less likely to report multimethod use (*P* = .028). Moreover, for each unit increase in positive parental norms about sex, adolescent women were nearly 8% more likely to report multimethod use (*P* = .014). Finally, the single environmental predictor examined in this study was significant, suggesting that adolescent women residing in cohesive neighborhood environments were nearly twice as likely to report multimethod use compared to those residing in less cohesive neighborhood environments (*P* = .041).

## 4. Discussion

The prevalence of multimethod use among this sample of adolescent women was nearly 35%, which is higher compared to rates reported elsewhere in the literature [21–23]. This may be a function of the way in which multimethod use was measured in this study, that is, by assessing contraceptive behaviors within the past 3 months rather than assessing concurrent use at last intercourse. Alternatively, the higher rate of multimethod use observed this sample of adolescent women receiving mental health treatment may also be a function of greater exposure and access to hormonal contraception through ongoing and regular contact with prescribing mental health and primary care professionals.

Another departure from the extant literature is that in this sample age was not significantly related to multimethod use (*P* = .818). This is not altogether surprising given that previous research may have not included a host of contextual influences in models examining multimethod use. Our results suggest that other factors are more important in determining whether female adolescents use multiple contraceptives rather than “being older,” and it is possible that, in previous studies, age may have been a proxy for some of these factors (e.g., having multiple partners). Specifically, our study identified factors associated with multimethod use at three contextual levels: individual, interpersonal, and environmental. First, at the individual level, adolescent women receiving psychological services who had a history of pregnancy were marginally less likely to report multimethod use than those without a history of pregnancy. The prior literature has consistently shown that four risk factor domains are associated with repeat teen pregnancy: disadvantaged socioeconomic background, single family household, psychological and emotional instability manifested in aggressive behavior, and exposure to chaotic environments and risky peer networks (e.g., violence and substance use) [32–38]. The current sample of adolescent women is characterized by a similar pattern of risk factors. Specifically, adolescent women in this study are particularly disadvantaged by virtue of their psychological and emotional disorders, with oppositional defiant disorder, ADHD, and conduct disorder being the most prevalent. These disorders are largely characterized by aggression, antisocial behaviors, and attention problems, all of which are documented risk factors for repeat teen pregnancy. 

Having multiple partners significantly predicted multimethod use, suggesting that although adolescent women may understand the risk associated with having multiple partners, those with single partners may feel unduly protected in their relationship with one sex partner, thus deciding against multimethod use. This is consistent with prior findings suggesting that condom use declines as relationships become more serious and exclusive [39–42]. In fact, the literature suggests that among adolescents, it takes less than one month for condom use to decline to levels observed among well-established, long-term relationships [43]. As such, education and prevention messages regarding multimethod use should particularly focus on adolescent women reporting monogamous relationships in addition to continuing to address the risk of having multiple partners.

Finally, at the individual level, condom use self-efficacy was a significant predictor of multimethod use. Previous research suggests that among adolescents who feel confident in their condom use skills, consistent condom use increases [44–46]; however, fewer studies have assessed whether self-efficacy predicts contraceptive use beyond condoms. Findings from one such study suggest that contraceptive self-efficacy, while not predicting condom use specifically, was a significant predictor of contraceptive use in general among adolescent females [47]. Even though the current study measured condom use self-efficacy rather than contraceptive self-efficacy, our results corroborate this previous finding. It may be that self-efficacy in one sexually related domain such as using condoms may generalize to other forms of contraception. 

Among interpersonal-level factors, an unexpected result suggests that, based on parent report, more frequent communication about sex with their adolescent daughters (including sex, condoms, contraception, HIV/AIDS, peer pressure, and sexual partner selection) was related to less multimethod use. Previous research has shown that parental communication about sex positively impacts sexual risk behaviors and often mitigates the effect of peer norms on adolescents' sexual risk behaviors [48–50]. Adolescents whose parents talk to them frequently and openly about sex also tend to use condoms more often and have fewer sexual partners thereby reducing their risk for STIs/HIV [48, 49, 51]. Yet, some research suggests that these protective effects may be mitigated for adolescents who are already sexually active [49]. Although this study measured the frequency and general content area of communication with adolescents, we did not measure the quality of the interactions nor the specific messages that were related to the adolescents within each content area. It may be possible that for our sample of adolescent women, who already experienced their sexual debut, some aspect of the communication process, either the style or the parental attitudes toward sex and contraception, could have contributed to the reversal of a protective effect. In fact, the current study also found a significant effect for parental norms about sex, suggesting that adolescents whose parents reported more positive attitudes toward sex were more likely to report multimethod use. Thus, it is feasible that more negative parental attitudes toward sex may interject a negative tone or more negative messages in parental communication to adolescents. As these negative messages then become reinforced with increased frequency in communication, a reduction in multimethod use may result.

Finally, neighborhood cohesion was a significant environmental-level factor associated with multimethod use suggesting that adolescent women living in a more cohesive environment, characterized by positive social contact and familiarity with neighbors, are nearly twice as likely to report multimethod use compared to adolescents residing in less cohesive environments. The mechanism by which neighborhood cohesion acts to increase condom use and other forms of contraception is unknown. Prior studies have suggested that a cohesive neighborhood environment may offer support to its adolescent residents in the form of caring, tolerance, and respect. In turn these characteristics may impact an adolescent's sense of self [51]. Research assessing the associations between social dynamics, sense of self, and health outcomes supports the assertion that social disorder can shape an individual's self-concept and subsequently can influence that individual's drug use and sexual risk taking [52, 53]. Among adolescent women coping with psychological disorders, the positive impact of neighborhood support and cohesion may be particularly salient. Further research is necessary to uncover the underlying mechanisms by which neighborhood cohesion is associated with various protective sexual health outcomes including condom use and multimethod use, particularly among adolescent women with psychological disorders.

### 4.1. Limitations

Several limitations to this study need to be acknowledged. First, the generalizability of the findings is limited to (a) adolescents meeting the specific criteria for inclusion in the randomized clinical trial and (b) the three geographic regions of the county from which the data are obtained. Second, data included in the current analyses were obtained through self-report and may reflect social desirability bias. Third, the multimethod use measure assessed condom use and one other birth control method. This could include a hormonal method such as the birth control pill or some other type of barrier method such as the diaphragm. The various possible combinations of multiple methods were not explored in this study as this would have significantly restricted the sample size available for analysis. Fourth, the multiple methods of contraceptive use were assessed over the previous 3 months with no assessment of concurrent use. Finally, length of relationship may play a role in adolescents' willingness to utilize contraception and should be assessed in future studies.

## 5. Conclusion

Understanding multilevel factors that predict multimethod contraceptive use among adolescent women with psychological disorders is crucial both in terms of clinical practice as well as in the design of STI/HIV and pregnancy prevention programs. Research should further investigate the specific factors that contribute to this protective behavior. Mental health as well as primary care providers should continue to focus on addressing issues of multimethod as well as dual-protection use with their female adolescent population. Furthermore, while public health professionals have historically focused on designing and implementing STI/HIV prevention programs separately from pregnancy prevention programs, given the high rates of STIs and unintended pregnancy among adolescent women, especially among those with compromised psychological states, it is advisable to design programs that target both epidemics concurrently through programs that incorporate modifiable factors at the individual, interpersonal, and environmental levels.

## Figures and Tables

**Table 1:** Characteristics of multimethod and non-multimethod users.

	Multimethod users (*n* = 100)		Non-multimethod users (*n* = 188)		*P*
	Mean (SD)	*N* (%)	Mean (SD)	*N* (%)	
*Sociodemographic factors*					
Age of adolescent	15.08 (1.22)		15.40 (1.27)		.038
Race					.680
Caucasian		30 (30.0)		59 (31.4)	
African American		58 (58.0)		99 (52.7)	
Other		3 (3.0)		3 (1.6)	
Ethnicity					.190
Hispanic		9 (9.0)		27 (14.4)	
Family income					.190
≤30 K/year		60 (64.5)		99 (56.3)	
>30 K/year		33 (35.5)		77 (43.8)	
Parent marital status					.161
Married/remarried		28 (28.0)		68 (36.2)	
Single parent		72 (72.0)		120 (63.8)	
Adolescent current sex partner					.001
Yes		72 (72.0)		94 (50.0)	
No		28 (28.0)		94 (50.0)	

**Table 2:** Bivariate associations between study variables and multimethod use and non-use.

	Multimethod users (*n* = 100)		Non-multimethod users (*n* = 188)		*P*
	Mean (SD)	*N* (%)	Mean (SD)	*N* (%)	
*Individual level factors*					
Condom use self-efficacy	47.34 (5.38)		43.47 (10.19)		.001
Multiple sex partners					.002
Yes		30 (30.0)		27 (14.7)	
No		70 (70.0)		157 (85.3)	.189
Pregnancy history					
Yes		12 (12.2)		34 (18.3)	
No		86 (87.8)		152 (81.7)	
*Interpersonal level factors*					
Sexual communication	8.45 (10.11)		10.11 (10.12)		.187
Parental norms about sex	26.56 (5.64)		24.28 (6.25)		.003
*Environmental level factors*					
Neighborhood cohesion					.007
Low cohesion		47 (48.0)		120 (64.5)	
High cohesion		51 (52.0)		66 (35.5)	

**Table 3:** Multivariable associations between individual-, interpersonal- and environmental level factors and multimethod use.

	Prevalence ratio	AOR^a^	95% conf. interval
Individual level factors			
Condom use self-efficacy	n/a	1.07	1.02–1.12
Multiple partners	0.49	2.87	1.27–6.48
Pregnancy history	1.49	0.41	0.17–1.00
Interpersonal level factors			
Sexual communication	n/a	0.97	0.94–0.99
Parental norms about sex	n/a	1.08	1.02–1.14
Environmental level factors			
Neighborhood cohesion	1.35	1.91	1.03–3.55

^a^Adjusted odds ratio using non-multimethod users as the referent category; models are controlling for adolescent age, race, ethnicity, household income, parent marital status, and adolescent current relationship status.

Model fit: *χ* ^2^ = 54.30; *P* = .001.
